# BAFF predicts immunogenicity in older patients with rheumatoid arthritis treated with TNF inhibitors

**DOI:** 10.1038/s41598-021-91177-4

**Published:** 2021-06-02

**Authors:** Borja Hernández-Breijo, Victoria Navarro-Compán, Chamaida Plasencia-Rodríguez, Ioannis Parodis, Johanna E. Gehin, Ana Martínez-Feito, Marta Novella-Navarro, Araceli Mezcua, David J. Warren, Pilar Nozal, Dora Pascual-Salcedo, Alejandro Balsa

**Affiliations:** 1 Immuno-Rheumatology Research Group, Hospital La Paz Institute for Health Research-IdiPAZ, Madrid, Spain; 2 Department of Rheumatology, La Paz University Hospital, Madrid, Spain; 3 Division of Rheumatology, Department of Medicine Solna, Karolinska Institutet, Stockholm, Sweden; 4 Department of Gastroenterology, Dermatology and Rheumatology, Karolinska University Hospital, Stockholm, Sweden; 5 School of Medical Sciences, Örebro University, Örebro, Sweden; 6 Department of Medical Biochemistry, Oslo University Hospital, Radiumhospitalet, Oslo Norway; 7 Faculty of Medicine, University of Oslo, Oslo, Norway; 8 Immunology Unit, La Paz University Hospital, Madrid, Spain; 9 Center for Biomedical Network Research on Rare Diseases (CIBERER U754), Madrid, Spain

**Keywords:** Rheumatic diseases, Autoimmune diseases, Immunotherapy

## Abstract

Immunogenicity related to treatment with TNF inhibitors (TNFi) is one of the causes for the decreased attainment of clinical response in patients with rheumatoid arthritis (RA). The B-cell activating factor (BAFF) may be playing a role in the development of immunogenicity. The objective of this study was to analyse the association of baseline concentration of serum B-cell activating factor (BAFF) with immunogenicity after 6 months of TNFi treatment. A total of 127 patients with RA starting a TNFi (infliximab, adalimumab, certolizumab pegol or golimumab) were followed-up for 6 months. Serum samples were obtained at baseline and at 6 months and anti-drug antibody (ADA) and BAFF concentrations were measured. Logistic regression models were employed in order to analyse the association between BAFF concentrations and immunogenicity. Receiver operating characteristic analysis was performed to determine the BAFF concentrations with a greater likelihood of showing immunogenicity association. At 6 months, 31 patients (24%) developed ADA. A significant interaction between the age and baseline BAFF concentration was found for the development of ADA (Wald chi-square value = 5.30; p = 0.02); therefore, subsequent results were stratified according to mean age (≤ / > 55 years). Baseline serum BAFF concentration was independently associated with ADA development only in patients over 55 years (OR = 1.51; 95% CI 1.03–2.21). Baseline serum BAFF ≥ 1034 pg/mL predicted the presence of ADA at 6 months (AUC = 0.81; 95% confidence interval (CI) 0.69–0.93; p = 0.001; positive likelihood ratio = 3.7). In conclusion, our results suggest that the association of BAFF concentration and immunogenicity depends on the patient’s age. Baseline serum BAFF concentration predicts the presence of ADA within 6 months of TNFi therapy in older patients with RA.

## Introduction

Tumour necrosis factor inhibitors (TNFi) are nowadays important therapeutic options for patients with rheumatic diseases, such as rheumatoid arthritis (RA). The efficacy of these therapies has been demonstrated in numerous clinical trials and observational studies. However, a substantial proportion of patients receiving TNFi experience inefficacy to this therapy due to the development of immunogenicity^1^.

B cells are central actors in the development of anti-drug antibodies (ADA). The B cell activating factor (BAFF) is a cytokine that belongs to the TNF ligand superfamily (member 13b), and is primarily produced by monocytes and neutrophils. BAFF is essential for B cell activation, differentiation and survival. These effects are mediated by the interaction between BAFF and its cell surface receptors (BAFF-R, TACI and BCMA), which activate the NF-κB signalling pathway to further trigger essential effector signals for the formation and maintenance of B cells^2^. Several studies recently showed that BAFF may be involved in this process by demonstrating a role for BAFF in the immunomodulatory mechanism of methotrexate (MTX) against immunogenicity associated to TNFi therapy. Glaesener et al. suggested that serum BAFF levels are not modified by MTX treatment in patients with juvenile idiopathic arthritis^3^. However, Bitoun et al. showed that antidrug antibody (ADA)-negative patients treated with TNFi and MTX showed higher baseline serum BAFF levels compared with ADA-positive patients^4^, suggesting that MTX might require high serum BAFF levels to impede the development of immunogenicity associated with TNFi therapy^4^. Nevertheless, the role of BAFF in the mechanism involved in the development of ADA and the influence of other factors in this relationship has not been thoroughly investigated. More studies are required in order to fully understand the role of BAFF as a potential predictor of increased risk of ADA development.

The main objective of this study was to investigate the association between serum BAFF levels and the development of immunogenicity after6months of TNFi treatment in patients with RA.

## Results

### Patients and disease´ characteristics

A total of 127 patients with RA starting TNFi (64 infliximab, 33 certolizumab pegol, 18 adalimumab and 12 golimumab) were included in this study. Patients starting etanercept were not included since, to our knowledge, no immunogenicity response to etanercept therapy has been demonstrated^5^. Demographics and clinical characteristics are summarized in Table 1. At 6 months of treatment, 31 (24%) patients were positive for serum ADA.

**Table 1:** Baseline patients’ characteristics.

	Total (n = 127)	ADA-negative (n = 96)	ADA-positive (n = 31)	p-value
**Clinical parameters**				
Age (years)	55 ± 14	56 ± 14	52 ± 15	0.1
Female	105 (83)	81 (84)	24 (77)	0.4
Disease duration (years)	9 (4–14)	8 (4–13)	9 (3–15)	0.7
Smokers	53 (42)	42 (44)	11 (35)	0.4
Body mass index (kg/m^2^)	25.9 ± 4.8	25.7 ± 4.9	26.5 ± 4.5	0.4
Baseline DAS28	5.1 ± 1.4	5.0 ± 1.2	5.5 ± 1.8	0.2
**Serological parameters**				
Seropositivity (RF and/or ACPA)	114 (90)	84 (87)	30 (97)	0.1
Baseline BAFF concentration (pg/mL)	933 ± 388	896 ± 347	1049 ± 481	0.1
**Therapy characteristics**				
Concomitant csDMARDs	115 (90)	90 (94)	25 (81)	**0.03**
MTX (only MTX or MTX + OD)	86 (68)	65 (68)	21 (68)	1.0
Dose of MTX (mg/week)	20.0 (15.0–25.0)	20.0 (15.0–25.0)	15.0 (10.0–20.0)	**0.006**
Only OD	29 (23)	25 (26)	4 (13)	0.1
Prednisone use	70 (55)	53 (55)	17 (55)	1.0
Previous TNFi therapy use	19 (14)	15 (16)	4 (13)	0.7

The table shows mean ± SD, median (IQR) or absolute number (percentage) for all patients included (n = 127). The results are also stratified by anti-drug antibody seropositivity. Significant statistical differences are noted in bold. p-value < 0.05 was considered statistically significant.

*RF* rheumatoid factor, *ACPA* anti-citrullinated peptide antibody, *BAFF* B cell activating factor, *csDMARDs* conventional synthetic disease-modifying anti-rheumatic drug, *DAS28* disease activity score-28, *TNFi* TNF inhibitor, *MTX* methotrexate, *OD* other csDMARDs.

Regarding to concomitant treatment, 12 patients (9%) were treated with TNFi in monotherapy, 86 patients (68%) with TNFi combined with MTX [with or without other csDMARDS (OD)], and 29 patients (23%) were treated with TNFi combined with OD comprising leflunomide, sulfasalazine or hydroxychloroquine. The use of concomitant csDMARDs was less frequent in ADA-positive patients than in ADA-negative patients (81% vs. 94%, respectively; p = 0.03). Moreover, patients who developed ADA received lower doses of concomitant MTX compared with ADA-negative patients (doses expressed as medians: 15 mg/week and 20 mg/week, respectively; p = 0.006).

### Association between BAFF concentration and immunogenicity after 6 months of TNFi treatment

Before analysing the association between baseline BAFF concentration and the immunogenicity, possible interactions among each patient’s characteristics (described in Table 1) and the baseline BAFF concentration for the development of immunogenicity were studied. A significant interaction between patients’ age and baseline BAFF concentration was found (Wald chi-square value = 5.30; p = 0.02) for the development of immunogenicity. No statistically significant interactions were found for other variables although an interacting trend between the dose of concomitant MTX and BAFF was observed (Wald chi-square value = 2.99; p = 0.08). Therefore, further analyses were stratified into two age groups, using the mean age of the study population (55 years) as the cut-off point to establish age-categories. Moreover, differences of patients’ characteristics between age groups were investigated (table S1). Patients over 55 years showed significant longer disease duration (p = 0.002) as well as higher concentrations of baseline BAFF than younger patients (1000 ± 378 pg/mL and 872 ± 389 pg/mL, respectively; p = 0.008).

According to the results of the univariable regression analyses, higher baseline DAS28 (OR 2.24; 95% CI 1.31–3.82) or the use of a lower dose of concomitant MTX (OR 0.87; 95% CI 0.76–0.98) were significantly associated with immunogenicity in younger patients). On the other hand, higher BAFF serum concentration (OR 1.41; 95% CI 1.11–1.79) was found significantly associated with immunogenicity in patients over 55 years. However, this association was not significant in younger patients (OR 0.96; 95% CI 0.82–1.13) (Table 2).

**Table 2:** Association between patients’ characteristics and the development of ADA at 6 m (univariable analysis), stratified by age.

Baseline patients’ characteristics	Age ≤ 55 years (n = 66)			Age > 55 years (n = 61)		
	OR	IC 95%	p-value	OR	IC 95%	p-value
Female	0.59	0.15–2.35	0.5	0.67	0.15–2.98	0.6
Disease duration (years)	0.99	0.91–1.09	0.9	1.03	0.97–1.10	0.3
Seropositivivity (RF and/or ACPA)*	–	–	–	0.80	0.08–8.40	0.8
Smokers	0.69	0.23–2.09	0.5	0.68	0.18–2.52	0.6
Body mass index (kg/m^2^)	1.01	0.91–1.14	0.8	1.07	0.93–1.21	0.3
Baseline DAS28	2.24	1.31–3.82	**0.003**	0.84	0.56–1.27	0.4
Previous TNFi treatment*	1.24	0.33–4.67	0.7	–	–	–
Dose of MTX (mg/week)	0.87	0.76–0.98	**0.03**	0.91	0.80–1.04	0.2
Baseline Prednisone	0.71	0.24–2.12	0.5	1.47	0.42–5.15	0.5
Baseline BAFF concentration (pg/mL)	0.96	0.82–1.13	0.6	1.41	1.11–1.79	**0.005**

The univariable logistic regression analysis included 127 patients. Odds ratio (OR) and 95% confidence interval (CI) were calculated. Significant statistical differences are noted in bold. p-value < 0.05 was considered statistically significant.

*RF* rheumatoid factor, *ACPA* anti-citrullinated peptide antibody, *DAS28* disease activity score-28, *TNFi* TNF inhibitor, *MTX* methotrexate, *BAFF* B cell activating factor.

*Result not provided due to complete separation.

**Figure 1:**
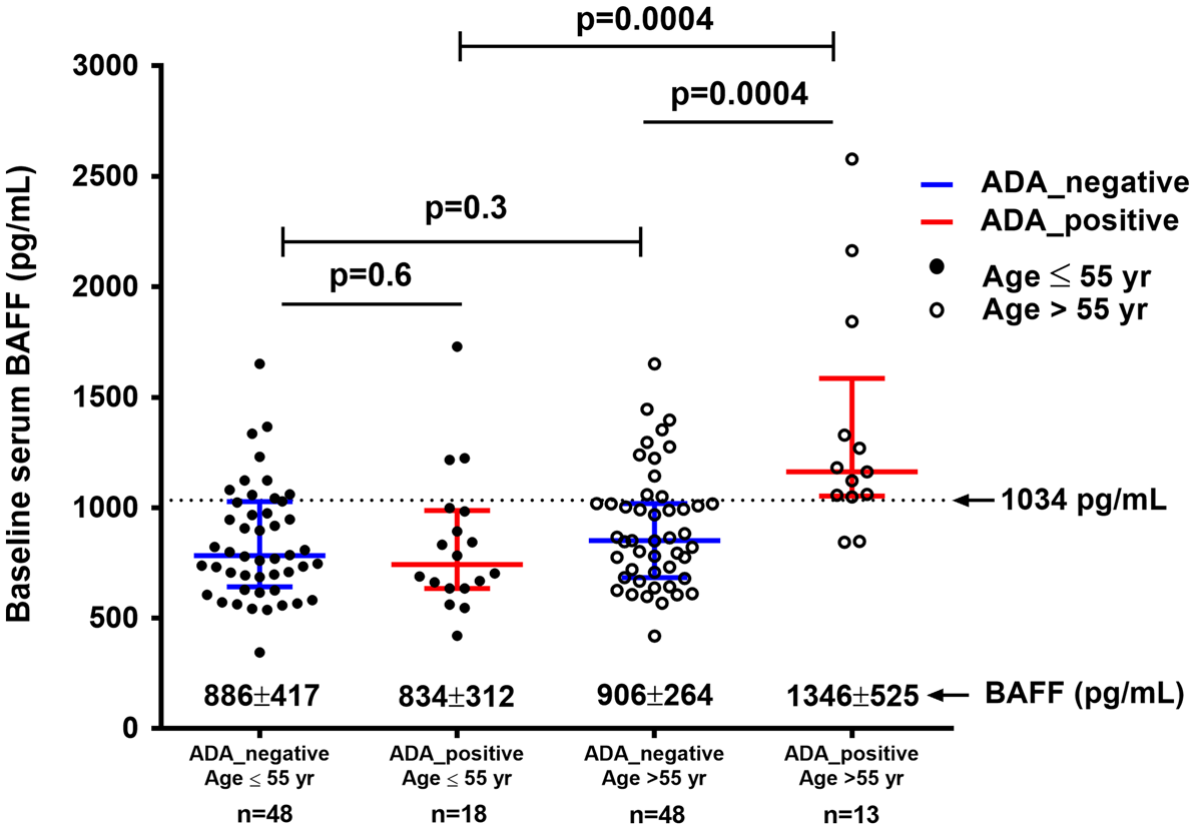
Baseline serum BAFF concentration (mean ± SD) stratified by groups based on ADA status and age (≤ / > 55 years). Dashed line indicated BAFF cut-off for predicting immunogenicity in patients older than 55 years. The results are shown as mean and standard deviation (SD). Comparisons were conducted using the Mann–Whitney U test. p-value < 0.05 was considered statistically significant.

In the group of patients younger than or equal to 55 years, baseline serum BAFF concentrations were similar regardless of ADA development (834 ± 312 pg/mL for ADA-positive vs. 886 ± 417 pg/mL for ADA-negative; p = 0.6). However, in the group of over 55 years old, ADA-positive patients showed significantly higher baseline serum BAFF concentrations than ADA-negative patients (1346 ± 525 pg/mL for ADA-positive vs. 906 ± 264 pg/mL for ADA-negative; p = 0.0004). Furthermore, within ADA-positive patients, baseline BAFF concentrations were higher in older than in younger patients (1346 ± 525 pg/mL for patients > 55 years vs. 834 ± 312 pg/mL for patients ≤ 55 years, p = 0.0004). These results are detailed in Fig. 1. In order to describe better these findings, a stratified analysis by age quartiles (age ≤ 45; 45 < age ≤ 55; 55 < age ≤ 65; age > 65) was performed. We found that BAFF concentration increased mainly in patients over 55 years (groups: 55 < age ≤ 65; age > 65) who developed ADA (table S2).

In addition, BAFF concentrations were analysed after 6 months of treatment. Within each age group, no statistical differences were found regarding BAFF concentrations at 6 months in relation to baseline (figure S1). However, BAFF concentrations at 6 months of therapy in ADA-positive patients over 55 years old were higher (1368 ± 611 pg/mL) compared to the rest of groups: younger ADA-positive patients (908 ± 243 pg/mL, p = 0.004), younger ADA-negative patients (888 ± 344 pg/mL, p = 0.001) and older ADA-negative patients (930 ± 275 pg/mL, p = 0.002) (figure S1).

Finally, a multivariable analysis including all the patients’ and disease´ characteristics with a p-value < 0.1 in the univariable analysis (DAS28, and the dose of concomitant MTX) was performed. These results revealed that baseline BAFF concentration (OR 1.51; 95% CI 1.03–2.21) was independently associated with ADA development after 6 months of treatment only for patients over 55 years. In contrast, this association was not found for younger patients: baseline BAFF concentration (OR 0.95; 95% CI 0.69–1.30) (Table 3). A bootstrap resampling matching learning technique (that involves taking random samples from the dataset with re-selection against which to evaluate the model) was performed to validate these results confirming this association (Supplemental file 1) and we found results in the same direction.

**Table 3:** Association between baseline BAFF concentration and the development of ADA at 6 m, stratified by age.

Baseline patients’ characteristics	Age ≤ 55 years (n = 66)			Age > 55 years (n = 61)		
	OR	IC 95%	p-value	OR	IC 95%	p-value
Baseline DAS28	2.83	1.28–6.25	**0.01**	0.82	0.45–1.50	0.5
Dose of MTX (mg/week)	0.89	0.77–1.04	0.1	0.88	0.74–1.03	0.1
Baseline BAFF concentration (pg/mL)	0.95	0.69–1.30	0.7	1.51	1.03–2.21	**0.03**

Logistic regression analysis adjusted by baseline DAS28 and the dose of concomitant MTX. Odds ratio (OR) and 95% confidence interval (CI) and p-values were calculated. Significant statistical differences are noted in bold. p-value < 0.05 was considered as statistically significant.

*DAS28* disease activity score-28, *MTX* methotrexate, *BAFF* B cell activating factor.

### Serum BAFF concentration threshold

**Figure 2:**
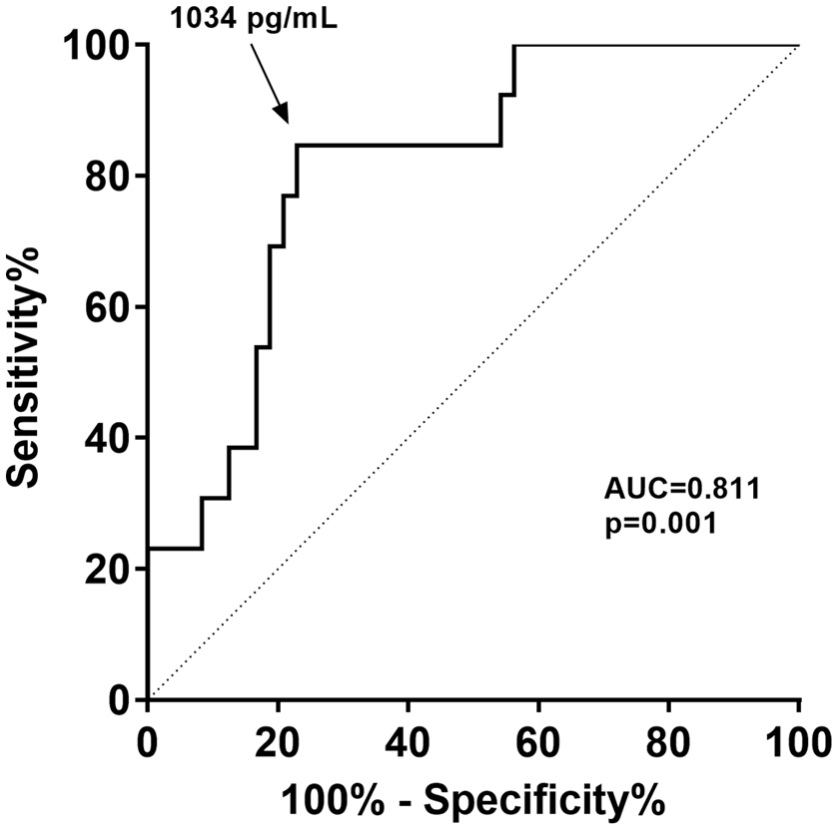
Receiver-operating characteristic curve for baseline serum BAFF concentration for prediction of the development of ADA at 6 m in patients older than 55 years. *AUC* Area Under the Curve. The serum BAFF concentration predictive cut-off (1034 pg/mL) was determined as the higher Youden index. p-value < 0.05 was considered statistically significant.

In order to determine an optimal threshold value for baseline serum BAFF concentration associated with the development of ADA within 6 months of TNFi treatment, a receiver operating characteristic analysis was performed in patients older than 55 years. The area under the curve was 0.81 (95% confidence interval (CI) 0.69–0.93; p = 0.001). We found that baseline serum BAFF concentration greater than or equal to 1034 pg/mL represented the concentration more likely to be associated with the development of ADA within 6 months of TNFi treatment (sensitivity = 85%, specificity = 77%, PPV = 59%, NPV = 93%), and this related to a positive likelihood ratio of 3.7 (Fig. 2). Threshold value is indicated in Fig. 1. This threshold value identified that 95% (37/39) of patients over 55 years with a baseline serum BAFF concentration lower than 1034 pg/mL did not develop ADA at 6 months of treatment with TNFi (table S3).

## Discussion

In this study, we aimed to investigate whether BAFF contributes to immunogenicity associated to TNFi treatment in patients with RA. In our cohort, we found an association between baseline serum BAFF concentrations and the development of ADA within 6 months of TNFi treatment but this finding was restricted to patients older than 55. Furthermore, baseline serum BAFF levels ≥ 1034 pg/mL seemed to be the best threshold to predict the presence of ADA at 6 months of TNFi treatment in patients over 55 years old. We demonstrate for the first time that the contribution of BAFF to the development of ADA depends on the age. Moreover, the dose of concomitant MTX seems to also interact with BAFF concentration.

Immunogenicity related to treatment with TNFi is one of the causes that hampers the achievement of clinical response in patients with RA^1^. The mechanisms underlying the development of ADA are not entirely clear. More studies are needed to understand why some patients develop ADA while others do not, and which factors amongst presence antibodies, antibody titres and other elements contribute to the loss of the biological action of TNFi, resulting in treatment failure. BAFF is believed to be one of the contributors on the development of immunogenicity^6, 7^. However, the role of BAFF in the mechanism behind the development of ADA has not been deeply investigated. Previous findings suggested that BAFF may have a role in the pathogenesis of RA^8, 9^. One possible explanation for the connection between BAFF and ADA development might be the role of BAFF in B cell maturation and survival, presumably also being the case for autoreactive B cell populations^10, 11^. We found ADA positive patients had higher serum BAFF concentrations prior to TNFi therapy initiation compared with patients who did not, but this association was restricted to patients who were older than 55 years. It has been demonstrated by previous publications that ADA positive patients show higher clinical activity prior to start TNFi^12–14^. This finding indirectly reflects that ADA positive patients could have a higher baseline production of cytokines and mediators of inflammation including BAFF. A plausible explanation for the association between the higher baseline BAFF concentration and the development of ADA found mainly in older patients could be the senescence of the B cell compartment, which leading to a diminished BAFF uptake by its receptors^2, 15–17^. Several studies have demonstrated that blood cell populations as well as the cytokine network can be modified by aging. Production of interferons by CD4^+^ and CD8^+^ T cells is enhanced in aged individuals, and it is connected with BAFF mobilisation and release from monocytes, macrophages and dendritic cells^11, 18–20^. Furthermore, immunosenescence during aging induces a decrease of B cells, especially plasma cells, starting at 40 years of age^16^. Nevertheless, despite this ostensible contradiction, the phenomenon of immunogenicity associated to TNFi treatment is not likely to be weakened by aging; supportive of this notion was a study by Paul et al. in which production of ADA was not altered in relation to increasing age^21^.

Different publications have demonstrated the usefulness of concomitant csDMARD use, particularly MTX, in preventing or decelerating immunogenicity and, consequently, promoting TNFi survival and TNFi efficacy^1, 22, 23^. The effect of concomitant MTX use in impeding immunogenicity receives increasing acknowledgement, with implications in clinical practice, but a proportion of patients still develop ADA despite use of MTX during TNFi therapy. Under further consideration, the dose of MTX could be an important factor^24, 25^. We herein observed that patients with RA who developed ADA at 6 months of treatment, significantly received lower doses of concomitant MTX compared with patients who did not develop ADA. Therefore, MTX had a significantly stronger dose–response effect on preventing the development of ADA.

Until now, the existence of a common denominator in the action mechanism between the preventive effect of MTX and the contributing role of BAFF has yet to be elucidated. MTX is known to lower numbers of B cells^3^, which partially explains the association between using lower doses of concomitant MTX and ADA development in the present study, but the impact of MTX on BAFF levels is less clear. A study has demonstrated reductions of BAFF levels upon MTX therapy^8^, which could possibly be supportive of a link between the two main observations in our study. However, other studies have shown associations between MTX use and elevated BAFF levels^26, 27^ or even no association^3^. Thus, deeper exploration is needed since current literature is inconclusive, but a link between MTX and BAFF levels related to the effect they exert on immunogenicity seems likely^4^. In conformity with this notion, we found in the present study a plausible interaction between the dose of MTX and BAFF levels, which suggests that BAFF may be involved in MTX mechanism to reduce the development of ADA. However, neither our originally study design nor the chosen sample size have allowed us to evaluate this effect in the different age groups, and should be addressed in a different study.

Due to the negative effect of immunogenicity on therapy efficacy, it is an urgent demand to find biomarkers that will predict the development of ADA. We found that a baseline serum BAFF concentration of 1034 pg/mL or greater was strongly associated to the development of ADA in patients of over 55 years of age. To our knowledge, this is the first time that BAFF concentration is reported as a predictive biomarker of ADA development at 6 months of TNFi treatment. The measurement of serum BAFF concentration is easy to perform and could be a useful tool for making more effective and personalized decisions for patients with RA before embarking on a TNFi treatment.

This study had some limitations, which need to be kept in mind when interpreting the results. The main limitation of the study was the low number of patients included. However, we would like to note that it is a pilot study with our rheumatoid arthritis (RA) cohort. Twenty-four percent of the patients developed anti-drug antibodies (ADA) at 6 months of treatment, and it is comparable to previous publications^1, 5, 28^. Moreover, due to the low number of patients included, the influence of concomitant MTX use on the association between BAFF concentrations and the development of ADA at 6 months of TNFi treatment could not be properly evaluated. Therefore, further studies will be performed to stratify the analyses by concomitant use of MTX. Another limitation would be that the limited number of patients forced the selection of the median for the cut-off for the age, instead of stratification by age tertile or quartile however, as it is shown in Table S2, this selection would be adequate because BAFF concentration increased in patients over 55 years who developed ADA. We would like to point out that this is a hypothesis generating study and therefore, further studies in larger populations will be needed to validate these results.

In conclusion, BAFF independently contributed to the development of ADA in patients with RA treated with TNFi, but this association is limited to older patients. Baseline serum BAFF concentration of 1034 pg/mL or greater before starting a TNFi may be a useful predictor of the development of immunogenicity in older patients with RA treated with this therapy. Additionally, BAFF concentration does not seem to be modulated by the treatment with TNFi.

## Methods

### Ethics approval and consent to participate

The Institutional Ethics Committee of the La Paz University Hospital approved the study (study code: PI-3244). The study was conducted according to the guidelines of the 1975 Declaration of Helsinki and complied with the Spanish legal requirements for the maintenance of confidential data. All the patients signed an informed consent document before inclusion.

### Patients

For this study, 127 patients with RA from the RA-Paz cohort were included. The RA-Paz cohort is a prospective, observational cohort comprising patients with RA, who have been initiated at biological DMARD treatment. All patients enrolled fulfilled the ACR/EULAR 2010 classification criteria for RA^29^, were over 18 years, had a moderate or high disease activity (DAS28 > 3.2), and fulfilled the criteria of the Spanish Society of Rheumatology recommendations regarding the use of biological therapies in RA^30^. Clinical data were systematically collected in a database by means of an electronic CRF at the Biologic Unit of La Paz University Hospital, and serum samples were frozen immediately and stored in the biobank of the Hospital. Disease activity was assessed using the *Disease Activity Score 28* (DAS28) at baseline (at the time of TNFi initiation, but before the first dose administration) and after 6 months of treatment. The patients were treated with TNFi (infliximab, adalimumab, certolizumab pegol or golimumab) and followed-up for 6 months. Serum samples were collected at baseline and at 6 months within a maximum of 24 h before the drug administration for subcutaneous TNFi, or immediately before intravenous infliximab infusions. ADA and BAFF levels were measured simultaneously in stored samples from baseline and 6 months.

### Measurement of anti-drug antibody levels

At 6 months of TNFi treatment, serum ADA levels were measured using bridging ELISA (infliximab, adalimumab, and golimumab)^1^. Anti-certolizumab antibodies were determined using a time-resolved fluorometric assay automated on the AutoDELFIA (PerkinElmer, Waltham, MA, USA) immunoassay platform^25^. All methods employed were drug-sensitive assays.

### Measurements of serum BAFF concentration

Serum BAFF levels were measured using the BAFF Human Instant ELISA Kit (R&D Systems, MN, USA) following the manufacturer's instructions. Briefly, an ELISA with an anti-human BAFF monoclonal coating antibody adsorbed onto microwells was used. Standards and samples were pipetted into the wells and any BAFF present was bound by the immobilized antibody. After washing away any unbound substances, an enzyme-linked polyclonal antibody specific for human BAFF was added to the wells. Following a wash to remove any unbound antibody-enzyme reagent, a substrate solution was added to the wells and colour developed in proportion to the amount of BAFF bound in the initial step. The reaction was terminated by addition of acid and absorbance was measured at 450 nm. A standard curve was prepared from seven human BAFF standard dilutions and human BAFF sample concentration was determined.

### Statistical analyses

First, descriptive analyses were performed for the demographic and clinical variables. The results are shown as mean and standard deviation (SD) or median (interquartile range, IQR) depending on normal distribution for continuous variables, and relative frequencies for categorical variables. The frequency data were compared using the Pearson chi-squared or Fisher’s exact tests. Comparisons of unpaired continuous data were conducted using the unpaired t-test or Mann–Whitney U test, depending on data distribution. Comparisons of paired continuous data were conducted using the paired t-test or Wilcoxon, depending on data distribution. For multiple comparisons, one-way ANOVA or Kruskal–Wallis test were used, depending on data distribution.

Second, associations between the development of immunogenicity within 6 months and clinical/serological variables were evaluated using univariable and multivariable logistic regression models, and data were presented as odds ratios, OR and 95% confidence intervals, CI. Any variable having a p-value < 0.1 at the univariable test was selected for the multivariable analysis. The presence of interactions between covariates was tested, and stratifications were performed for significant interactions (p < 0.05). In case of no interaction, the model was later adjusted for these covariates. Finally, receiver operating characteristic (ROC) analysis was performed to determine the baseline serum BAFF concentration that is more likely associated with the development of ADA throughout 6 months of TNFi treatment. This predictive cut-off was determined as the higher Youden index.

In order to validate the obtained results, a Bootstrap resampling machine learning technique (that involves taking random samples from the dataset with re-selection against which to evaluate the model) was performed. The total sample was partitioned into training (80%) and validation (20%) groups. Then, five predictive models were estimated and compared:

Model 1: ADA6m ~ baseline DAS + baseline BAFF + Age + baseline_BAFF:Age.

Model 2: ADA6m ~ baseline DAS + baseline BAFF + Age.

Model 3: ADA6m ~ baseline DAS + Age.

Model 4: ADA6m ~ baseline DAS.

Model 5: ADA6m ~ Age.

For each model, training data were adjusted to a Generalized Linear Model (binary response). To compared models, a bootstrapping matching learning technique (B = 500) was used to estimate a global ROC value for each model and the 95% confidence interval. These analyses were performed using R software v3.5.3 (2019-03-11) (https://www.r-project.org/) and the interface RStudio v1.2.5042, 2009–2020 Inc. The package used to performed graphs was ggplot2 v3.3.2, the package used to perform GLM and boostrap was Caret v6.0–86, the package used to perform the AUC analysis was pROC.

A p-value < 0.05 was considered statistically significant. The Statistical Package for the Social Sciences version 24 (SPSS, Chicago, IL, USA) was used for the analyses. The GraphPad Prism version 7 (GraphPad Software, San Diego, CA, USA) was used to prepare the graphs.

## Supplementary Information

Supplementary Information 1.

Supplementary Information 2.

Supplementary Information 3.

Supplementary Information 4.

Supplementary Information 5.

## Data Availability

B.H-B and A.B had full access to all of the data in the study and take responsibility for the integrity of the data and the accuracy of the data analysis. The datasets generated during and/or analysed during the current study are available from the corresponding author on reasonable request.
