# Supplementary material for: BAFF predicts immunogenicity in older patients with rheumatoid arthritis treated with TNF inhibitors
Source: Sci Rep. 2021 Jun 2;11:11632. doi: 10.1038/s41598-021-91177-4 (PMC8172642; doi:10.1038/s41598-021-91177-4)
Supplement: Supplementary file 1 — Supplementary Information 1. [file 41598_2021_91177_MOESM1_ESM.pdf]

Internal validation of the association between baseline BAFF concentration and the development of ADA at 6m, stratified by age. A Bootstrap resampling matching learning technique (that involves taking random samples from the dataset with re-selection against which to evaluate the model) was performed. The total sample was partitioned into training (80%) and validation (20%) group. Then, five predictive models were estimated and compared:

Model 1: ADA6m ~ baseline DAS + baseline BAFF + Age + baseline_BAFF:Age
Model 2: ADA6m ~ baseline DAS + baseline BAFF + Age
Model 3: ADA6m ~ baseline DAS + Age
Model 4: ADA6m ~ baseline DAS
Model 5: ADA6m ~ Age

For each model training data were adjusted to a Generalized Linear Model (binary response). To compared models, a bootstrapping matching learning technique (B=500) was used to estimated a global ROC value for each model and the 95% confidence interval. The graph comparing these ROC values and 95% CI is showed below:

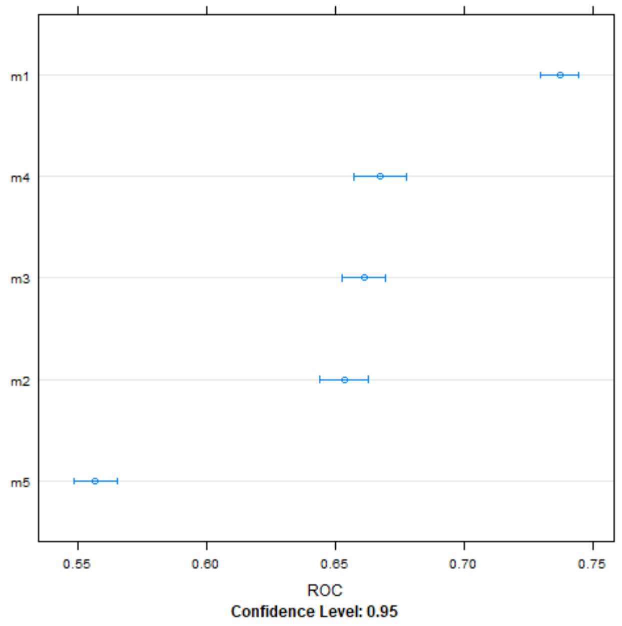

We found that the model including the interaction between baseline BAFF and the age (Model 1: ADA6m ~ baseline DAS + baseline BAFF + Age + baseline_BAFF:Age) showed higher predictive ability than the models who did not consider this interaction. Furthermore, by applying Model 1 we found results in the same direction showed in the manuscript:

**Generalized Linear Model**<br>**Bootstrap sampling; B=500**	**Age≤55years (n:57)**		**Age>55years (n:45)**	
	**OR**	**p-value**	**OR**	**p-value**
Baseline BAFF concentration (pg/mL)	0.92	0.4	1.61	**0.009**

Then, we analysed the predictive ability of the validation group (20% of the sample). We found that the validation group was predictive by showing: AUC= 0.645; 95% CI=0.406-0.883; sensitivity=50%; specificity=79%; PPV=43%; NPV=83%.

Significant statistical differences are noted in bold. p-value<0.05 was considered as statistically significant. DAS28, disease activity score-28; BAFF, B cell activating factor.
