# Supplementary material for: BAFF predicts immunogenicity in older patients with rheumatoid arthritis treated with TNF inhibitors
Source: Sci Rep. 2021 Jun 2;11:11632. doi: 10.1038/s41598-021-91177-4 (PMC8172642; doi:10.1038/s41598-021-91177-4)
Supplement: Supplementary file 2 — Supplementary Information 2. [file 41598_2021_91177_MOESM2_ESM.pdf]

SUPPLEMENTAL MATERIAL

BAFF predicts immunogenicity in older patients with rheumatoid arthritis treated with TNF inhibitors

Borja Hernández-Breijo, Victoria Navarro-Compán, Chamaida Plasencia-Rodríguez, Ioannis Parodis, Johanna E. Gehin, Ana Martínez-Feito, Marta Novella-Navarro, Araceli Mezcua, David J. Warren, Pilar Nozal, Dora Pascual-Salcedo, Alejandro Balsa

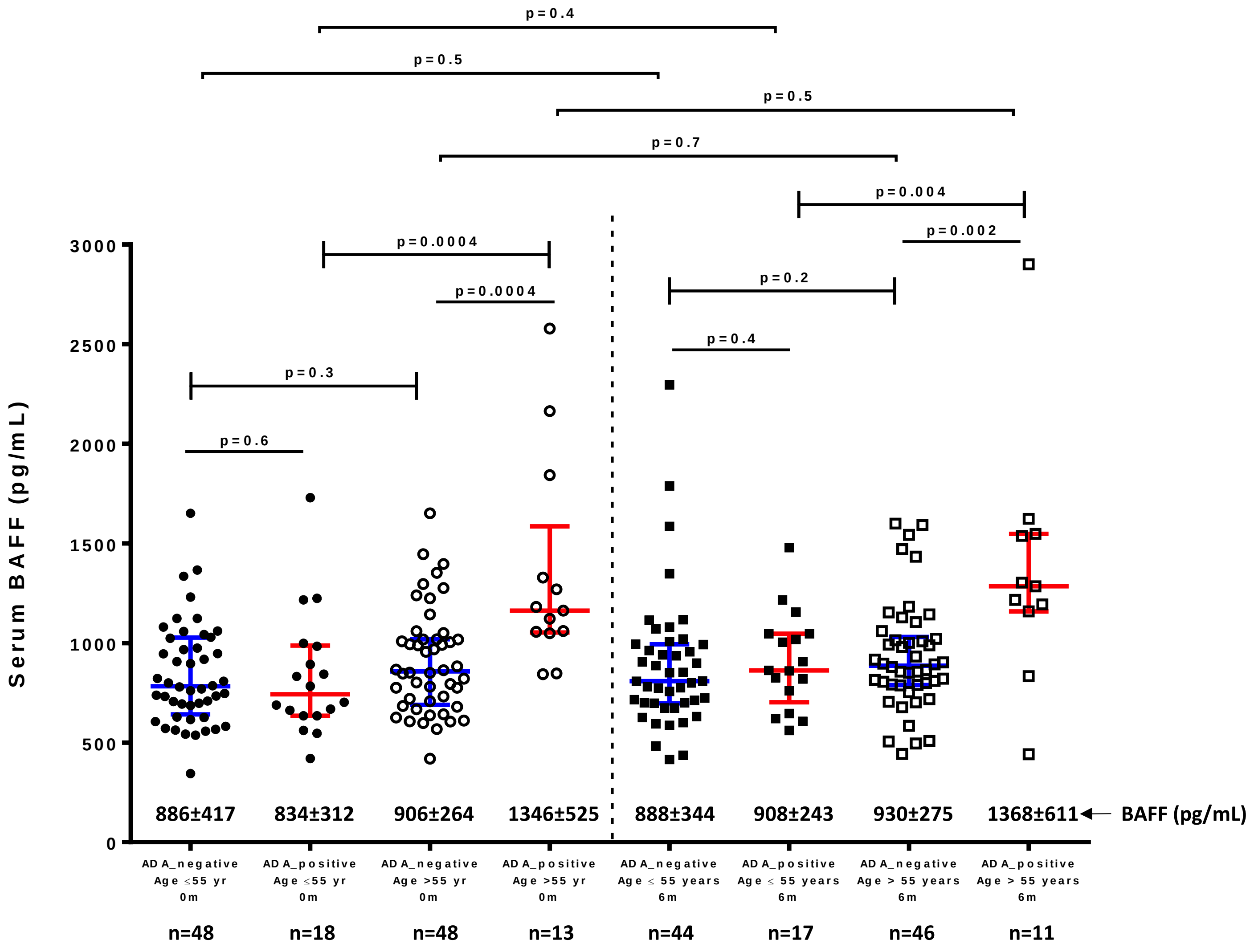

Figure S1: Serum BAFF concentration (mean±SD) measured at the baseline and 6m of TNFi treatment, stratified by groups based on ADA status and age (≤/>55years). The 4 columns on the left represent the serum BAFF concentrations at the baseline, and the 4 columns on the right represent the serum BAFF concentrations after 6m. The results are shown as mean and standard deviation (SD). Comparisons of unpaired continuous data were conducted using Mann-Whitney U test and comparisons of paired continuous data were conducted using the Wilcoxon test. p-value<0.05 was considered statistically significant.
