# Supplementary material for: BAFF predicts immunogenicity in older patients with rheumatoid arthritis treated with TNF inhibitors
Source: Sci Rep. 2021 Jun 2;11:11632. doi: 10.1038/s41598-021-91177-4 (PMC8172642; doi:10.1038/s41598-021-91177-4)
Supplement: Supplementary file 3 — Supplementary Information 3. [file 41598_2021_91177_MOESM3_ESM.docx]

|  | **Total**  **(n=127)** | **Age≤55yr**  **(n=66)** | **Age>55yr**  **(n=61)** | **p-value** |
| --- | --- | --- | --- | --- |
| **Clinical parameters** | | | | |
| Female | 105 (83) | 55 (83) | 50 (82) | 0.8 |
| Disease duration (years) | 9 (4-14) | 7 (3-11) | 11 (6-18) | **0.002** |
| Smokers | 53 (42) | 30 (45) | 23 (38) | 0.4 |
| Body mass index (kg/m2) | 25.9±4.8 | 25.6±4.9 | 26.2±4.7 | 0.5 |
| Baseline DAS28 | 5.1±1.4 | 5.2±1.3 | 5.1±1.5 | 0.8 |
| **Serological parameters** | | | | |
| Seropositivity (RF and/or ACPA) | 114 (90) | 57 (86) | 57 (93) | 0.2 |
| ADA positive | 31 (24) | 18 (27) | 13 (21) | 0.4 |
| Baseline BAFF concentration (pg/mL) | 933±388 | 872±389 | 1000±378 | **0.008** |
| **Therapy characteristics** | | | | |
| Concomitant csDMARDs | 125 (90) | 61 (92) | 54 (88) | 0.4 |
| MTX (only MTX or MTX+OD) | 94 (68) | 46 (70) | 40 (66) | 0.6 |
| Dose of MTX (mg/week) | 20.0 (12.5-22.5) | 20.0 (15.0-20.0) | 20.0 (12.5-25.0) | 0.5 |
| Only OD | 31 (22) | 15 (23) | 14 (23) | 1.0 |
| Prednisone use | 77 (55) | 37 (56) | 33 (54) | 0.8 |
| Previous TNFi therapy use | 19 (14) | 13 (20) | 6 (10) | 0.1 |

**Table S1: Baseline patients’ characteristics.** The table shows mean±SD, median (IQR) or absolute number (percentage) for all patients included (n=127). The results are also stratified by age (≤55/>55 years). Significant statistical differences are noted in bold. p-value<0.05 was considered statistically significant. RF, rheumatoid factor; ACPA, anti-citrullinated peptide antibody; BAFF, B cell activating factor; csDMARDs, conventional synthetic disease-modifying anti-rheumatic drug; DAS28, disease activity score-28; TNFi, TNF inhibitor; MTX, methotrexate; OD, other csDMARDs.
