# Supplementary material for: BAFF predicts immunogenicity in older patients with rheumatoid arthritis treated with TNF inhibitors
Source: Sci Rep. 2021 Jun 2;11:11632. doi: 10.1038/s41598-021-91177-4 (PMC8172642; doi:10.1038/s41598-021-91177-4)
Supplement: Supplementary file 4 — Supplementary Information 4. [file 41598_2021_91177_MOESM4_ESM.docx]

**Table S2: Baseline serum BAFF concentration according to age quartiles, stratified by ADA seropositivity.** Mann-Whitney U tests were applied and p-values were calculated for each age group comparing between ADA positivity groups. Kruskal-Wallis test was performed to compare baseline BAFF concentration among age quartiles for each ADA positivity groups. **(*p<0.05; **p<0.01)** indicate significant differences between each age quartile and Q1. Significant statistical differences are noted in bold. p-value<0.05 was considered as statistically significant. ADA, anti-drug antibodies; BAFF, B cell activating factor.

| **Baseline BAFF concentration (pg/mL)** | **All patients**  **(n=127)** | **ADA negative**  **(n=96)** | **ADA positive**  **(n=31)** | **p-value** |
| --- | --- | --- | --- | --- |
| age≤45 (years), n=31 (24%) (Q1) | 762±186 | 782±200 (n=22) | 712±145 (n=9) | 0.7 |
| 45<age≤55 (years), n=35 (28%) (Q2) | 969±488 | 974±525 (n=26) | 957±391 (n=9) | 0.9 |
| 55<age≤65 (years), n=29 (23%) (Q3) | 952±320 | 876±294 (n=21) | 1152±316 (n=8) **(*)** | **0.01** |
| age>65 (years), n=32 (25%) (Q4) | 1044±423 **(**)** | 930±240 (n=27) | 1658±674 (n=5) **(**)** | **0.002** |
