# Supplementary material for: BAFF predicts immunogenicity in older patients with rheumatoid arthritis treated with TNF inhibitors
Source: Sci Rep. 2021 Jun 2;11:11632. doi: 10.1038/s41598-021-91177-4 (PMC8172642; doi:10.1038/s41598-021-91177-4)
Supplement: Supplementary file 5 — Supplementary Information 5. [file 41598_2021_91177_MOESM5_ESM.docx]

**Table S3: Association between the baseline serum BAFF concentration threshold and the development of ADA at 6m, stratifying by age.** Pearson chi-squared was performed and p-values were calculated. Significant statistical differences are noted in bold. p-value<0.05 was considered as statistically significant. ADA, anti-drug antibodies; BAFF, B cell activating factor.

|  | **Age≤55years**  **(n=66)** | | | **Age>55years**  **(n=61)** | | |
| --- | --- | --- | --- | --- | --- | --- |
|  | **ADA negative**  **(n=48)** | **ADA positive**  **(n=18)** | **p-value** | **ADA negative**  **(n=48)** | **ADA positive**  **(n=13)** | **p-value** |
| Baseline BAFF<1034pg/mL (n=91) | 37 | 15 | 0.6 | 37 | 2 | **10^-5^** |
| Baseline BAFF≥1034pg/mL (n=36) | 11 | 3 |  | 11 | 11 |  |
